# Gene expression analysis of glioblastomas identifies the major molecular basis for the prognostic benefit of younger age

**DOI:** 10.1186/1755-8794-1-52

**Published:** 2008-10-21

**Authors:** Yohan Lee, Adrienne C Scheck, Timothy F Cloughesy, Albert Lai, Jun Dong, Haumith K Farooqi, Linda M Liau, Steve Horvath, Paul S Mischel, Stanley F Nelson

**Affiliations:** 1 Department of Human Genetics, David Geffen School of Medicine, University of California Los Angeles, Los Angeles, California 90095-7088 USA; 2 The Barrow Neurological Institute, St. Joseph's Hospital and Medical Center, Phoenix, Arizona 85013 USA; 3 Department of Neurology, David Geffen School of Medicine, University of California Los Angeles, Los Angeles, California 90095-1769 USA; 4 Department of Neurosurgery, David Geffen School of Medicine, University of California Los Angeles, Los Angeles, California 90095-6901 USA; 5 Department of Biostatistics, David Geffen School of Medicine, University of California Los Angeles, Los Angeles, California 90095-1772 USA; 6 Jonsson Comprehensive Cancer Center, University of California Los Angeles, Los Angeles, California 90095-1781 USA

## Abstract

**Background:**

Glioblastomas are the most common primary brain tumour in adults. While the prognosis for patients is poor, gene expression profiling has detected signatures that can sub-classify GBMs relative to histopathology and clinical variables. One category of GBM defined by a gene expression signature is termed ProNeural (PN), and has substantially longer patient survival relative to other gene expression-based subtypes of GBMs. Age of onset is a major predictor of the length of patient survival where younger patients survive longer than older patients. The reason for this survival advantage has not been clear.

**Methods:**

We collected 267 GBM CEL files and normalized them relative to other microarrays of the same Affymetrix platform. 377 probesets on U133A and U133 Plus 2.0 arrays were used in a gene voting strategy with 177 probesets of matching genes on older U95Av2 arrays. Kaplan-Meier curves and Cox proportional hazard analyses were applied in distinguishing survival differences between expression subtypes and age.

**Results:**

This meta-analysis of published data in addition to new data confirms the existence of four distinct GBM expression-signatures. Further, patients with PN subtype GBMs had longer survival, as expected. However, the age of the patient at diagnosis is not predictive of survival time when controlled for the PN subtype.

**Conclusion:**

The survival benefit of younger age is nullified when patients are stratified by gene expression group. Thus, the main cause of the age effect in GBMs is the more frequent occurrence of PN GBMs in younger patients relative to older patients.

## Background

Glioblastoma Multiforme (GBM) persists as one of the most lethal forms of human cancer with a median survival of 12 to 14.6 months for patients who receive the latest surgical, radiation, and chemotherapy treatment [1]. While increased efficacy of some chemotherapy drugs is evident, the prognosis remains dismal [2]. Despite the number of patients diagnosed with primary malignant brain and central nervous system tumours is small (relative to other cancer types i.e. 1.35% of all primary malignant cancers, U.S.), the morbidity and mortality of these patients remains severe [3].

Gliomas are primarily identified by histological features established by the grading scale of the World Health Organization (WHO). Prominent features include necrosis, nuclear mitotic activity, vascular proliferation, and cellular atypia [4]. In clinical oncology, the primary method for identifying survival factors in high-grade gliomas has been through Cox proportional hazards models. Covariates such as histological classification, performance status, and patient age at the time of surgery are particularly useful to predict patient outcomes [5,6]. Numerous studies have indicated that lower tumour WHO grade and younger patient age are the most prominent indicators of longer survival. Of patients diagnosed with a malignant glioma, younger patients have a disproportionately higher likelihood of being diagnosed with a lower grade glioma relative to older patients. However, a frequently observed phenomenon is that even among patients with the same high grade of glioma, younger patients tend to have a longer survival time. The reason for this survival advantage has not been clear [7].

Our group in 2004 used a heterogeneous group of high-grade gliomas that were categorized as WHO grade III and grade IV to identify molecularly classifiable groups irrespective of histologic typing and correlated with survival [8]. Despite this histological heterogeneity, these gliomas could be successfully stratified into at least three molecularly-defined glioma subgroups whose classification predicted patient survival. In particular, the HC1A tumours were identified as a good survival group, which were categorized by the high expression of genes typically expressed during neuronal development. The remaining groups HC2A and HC2B were characterized by mitotic and extra-cellular matrix related genes, respectively, and both demonstrated poor survival prognoses. Recently, the increased generation of whole genome microarray expression data has supported the gene expression signatures of the three previously identified GBM subgroups and the correlation with patient survival [9,10]. Phillips and colleagues suggested the following terms to be more descriptive of the cellular characteristics: 'ProNeural' (PN) for HC1A, 'Proliferative' (Pro) for HC2A, and 'Mesenchymal' (Mes) for HC2B, which we adopt here [9].

In order to investigate the robustness and generality of the gene expression-based groupings of GBMs, we have aggregated the publicly available genome scale expression data of histologically defined GBMs. Only samples performed on the Affymetrix platforms were included for joint analysis and analytical simplicity. In total, 181 GBMs were identified from the published literature for which Affymetrix microarrays were performed and CEL files were available. For many of these samples, survival and patient age were available [8-13]. In order to have sufficient numbers of samples to explore the correlation of expression signatures with age in GBMs, we have also performed gene expression analysis of an additional 86 GBMs in addition to this publicly available dataset. From this combined analysis, we demonstrate that about 86% of all tumour biopsies classify strongly into one of the three molecular subgroups as defined by the available gene list from Freije *et al*., and for about 11%, there is strong evidence of a novel group of GBMs that share expression features of Pro and Mes subtypes from the bulk biopsy analysis. Further, we investigated if there was a correlation between the molecular subtypes in relation to age of the patient at time of diagnosis and survival. We find that PN tumours are substantially more commonly diagnosed in younger patients, and that there is no survival advantage of age independent of gene expression classification. In other words, the beneficial effect of younger age in patients diagnosed with GBM is entirely due to the observation that for yet undefined reasons, younger adult patients develop PN type GBMs more commonly than older patients.

## Results

### Agglomeration of publicly available high-grade glioma microarray data confirms the presence of four molecularly distinct prognostic groups

**Figure 1:**
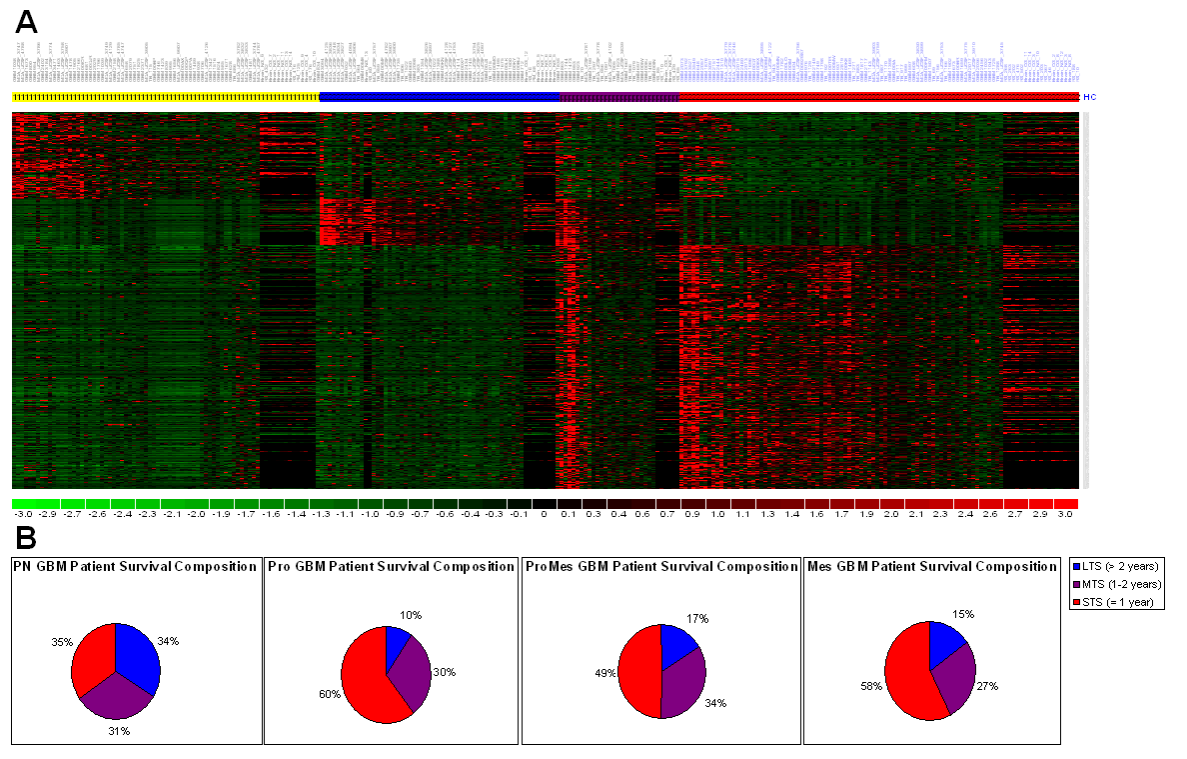
**Gene expression-based profiles related to survival duration across GBM tumours**. A. 377 probesets were used to classify the GBMs available on the U133A and U133 Plus 2.0 arrays, while 177 probesets were used to classify the tumours available on the U95Av2 arrays. The four glioblastoma types: PN: ProNeural (yellow, n = 77), Pro: Proliferative (blue, n = 61), ProMes: Proliferative-Mesenchymal (purple n = 29), and Mes: Mesenchymal (red, n = 100). B. Percent composition of Long (LTS), Medium (MTS), and Short (STS) term survival across the three glioma gene-signature tumour types. Over one-third of PN-classified tumour patients survive over 2 years, while over half of Pro, ProMes, and Mes classified tumour patients succumb in less than 1 year.

In our previous work in characterizing and exploring unrecognized subtypes of GBMs using genome-scale expression analysis, our laboratory was able to develop clear evidence of different gene expression signatures in GBMs, and further demonstrated that the HC1A subtype had prolonged patient survival relative to the HC2A and HC2B types [8]. Several groups have now published large gene expression analyses on GBMs that permit a more robust meta-analysis and exploration of the genomic landscape of GBMs. Through the efforts of several groups and the sharing of raw microarray data, the dataset available for addressing questions of gene expression status of individual genes and signatures has expanded to 267 glioblastomas (Table 1) and are organized and made available as a unified dataset here. This larger scale data permits exploration and testing of hypotheses generated in the initial microarray studies. We originally hypothesized that there were at least three subtypes of molecularly-distinct gliomas. With the accumulated genome-scale gene expression data, we initially thought that additional gene expression signature subtypes would become more evident. However, over 97% of our additional tumour samples (from multiple institutions) continue to bin clearly into one of the initially defined subtypes (Fig. 1A). A small set of tumours (11%) show evidence of a new category which has expression features of both HC2A and HC2B. The existence of the HC1A, HC2A and HC2B subgroups was corroborated by Phillips *et al*. independently [9] and identified within previously published data [13]. The Phillips *et al*. group suggested descriptive names for the gene expression signature based GBMs with HC1A named ProNeural (PN), HC2A named Proliferative (Pro), and HC2B named Mesenchymal (Mes). We indicate here that a group of GBMs with both Pro and Mes gene expression signatures exists which we name "ProMes"(Figure 1A).

**Table 1:** Combined Data Sources

Studies	No. GBM IV	Set	Array	PMID	GEO Accession	HC Class Distribution
Freije *et al*. [8]	46	0	U133A	15374961	GSE4412	PN (19/46)
						Pro (11/46)
						ProMes (2/46)
						Mes (14/46)
Phillips *et al*. [9]	55	2,3	U133A	16530701	GSE4271	PN (18/55)
						Pro (19/55)
						ProMes (4/55)
						Mes (14/55)
Rich *et al*. [10]	31	4	U133A	15899794	NA	PN (2/31)
						Pro (6/31)
						ProMes (8/31)
						Mes (15/31)
Mischel *et al*. [11]	2	7	U95Av2	12700671	NA	PN (1/2)
						Pro (1/2)
						ProMes (0/2)
						Mes (0/2)
Shai *et al*. [12]	19	8	U95Av2	12894235	NA	PN (3/19)
						Pro (2/19)
						ProMes (4/19)
						Mes (10/19)
Nutt *et al*. [13]	28	6	U95Av2	12670911	NA	PN (10/28)
						Pro (7/28)
						ProMes (2/28)
						Mes (9/28)
UCLA New	28	1	U133A	New	New	PN (11/28)
						Pro (3/28)
						ProMes (1/28)
						Mes (13/28)
UCLA New	27	1	U133 2.0	New	New	PN (7/27)
						Pro (3/27)
						ProMes (5/27)
						Mes (12/27)
Barrow New	31	5	U133A	New	New	PN (6/31)
						Pro (9/31)
						ProMes (3/31)
						Mes (13/31)

Total	267					

All of the microarray platforms can be used to identify the PN, Pro, Mes and ProMes subgroups of GBMs. Thus, we were able to merge data from the three most recent Affymetrix genome-scale expression arrays for humans. In terms of the gene voting compatibility between different microarray platforms, we calculate that the percentages of probesets which vote samples into their HC categories are quite similar. For the PN samples an average of 52%, 54%, and 47% of the PN probesets vote these tumours as PN across the U95Av2, U133A, and U133 Plus.2.0 arrays, respectively. The Pro samples also vote with an average of 72%, 69%, and 92% of the Pro probesets contributing to the positive votes among the three arrays. The ProMes category was identifiable from data from each of the three platforms with an average of 58% and 43% (Pro and Mes probesets, respectively), 64% and 45%, and 85% (only n = 3 U133 Plus 2.0 "Pro" samples) and 55% of their probesets. Finally, Mes samples vote with an average of 57%, 62%, and 51% of the Mes probesets on all three array types. Each sample voted over 47% of its category's probesets when voting into its respective HC class. Thus, even though differing sets of probes were used to identify the subgroups from the array platforms, the groups themselves were reliably identified.

The patient survival durations of each gene expression group confirms the prognostic utility of the gene signature-based predictor (Fig. 1B). PN GBM patients (n = 77) have a mean survival of 2.16 years (median = 1.4 years) while Pro (n = 61), Mes (n = 100), and ProMes (n = 29) GBM patients have a mean survival of 1.05 years (median = 283 days), 1.20 years (median = 322 days), and 1.44 years (median = 385 days), respectively. Moreover, only 35% of PN patients survive less than 1 year from the date of surgical resection, while about 60% of Pro, 49% of ProMes, and 58% of Mes patients succumb to their malignancies within the first year after resection. Part of the reason for the low survival is likely due to the lack of homogenous treatment across institutions over a period of several years. However, we note that among patients who received current standard of care (e.g. combined high dose radiotherapy, aggressive surgery, and Temozolomide (N = 36)), only 44% of these patients were alive at 12 months post diagnosis and only 39% (n = 14/36) survive up to or beyond 14.6 months. Thus, differences in therapeutic efficacy from the various studies included here are unlikely to alter the fundamental conclusions and that therapy is a minor contributor to the effects measured in terms of patient survival. The findings from this expanded dataset firmly corroborate the importance of gene signature based classification in categorizing glioblastomas into prognostically meaningful molecular groups. While there is little difference in survival between Pro, Mes, and ProMes tumour carriers, all have dramatically shorter patient survival times than the PN GBM patients. For the subset of the patients where treatment data were available, there were no differences in treatments attempted in the classification groups to account for the difference in survival length of the patients.

### Molecular Classification & Age Analysis

For adult patients who are diagnosed with GBM, younger age at diagnosis is a strong predictor of longer patient survival. The mechanism for this observation has not been clear, and in the study by Freije *et al*., age and PN tumour type were clearly correlated [8]. With the larger group of patients and GBM tumour biopsies available from the published work of several groups and new data reported here, we explore the relationship between the age of the patient, survival, and gene expression signature-based subtype of GBM. We sought to determine the relative importance of the PN subtype as compared to age of the patient. For these analyses, 28 GBM samples were removed from the analysis as no available age data were provided from the published data.

**Figure 2:**
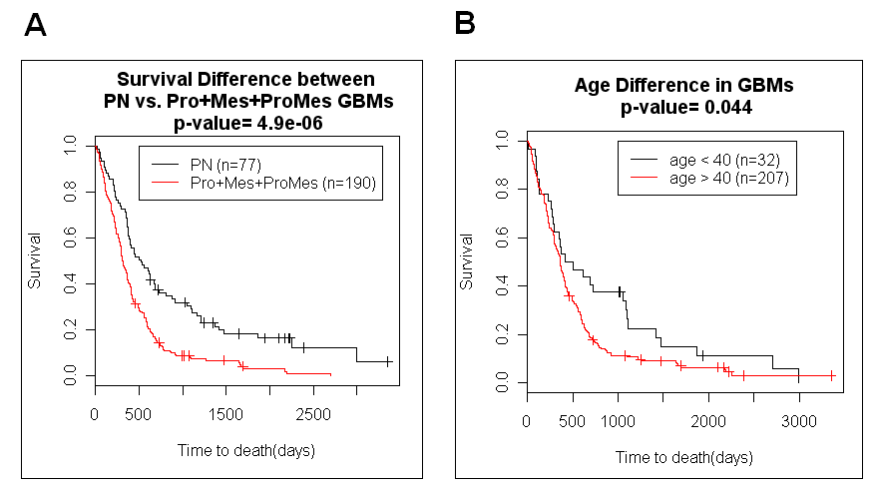
**Kaplan-Meier analysis of GBM patient survival according to predictive gene expression signatures and age alone**. A. PN GBM patients vs. non-PN GBM patients. Molecularly categorized GBM patient survival comparison shows PN GBMs survive longer than non-PN GBMs (Pro, Mes, and ProMes). PN GBM patient survival (mean = 2.2 years, median = 1.4 years) vs. non-PN GBM patient survival (mean = 1.2 years, median = 0.9 years). B. GBM patient age difference for survival: younger than age 40 vs. older than age 40. GBM patients younger than age 40 survive longer than GBM patients older than age 40. GBM age < 40 patient survival (mean = 2.1 years, median = 1.3 years) vs. GBM age > 40 patient survival (mean = 1.4 years, median = 0.9 years).

**Figure 3:**
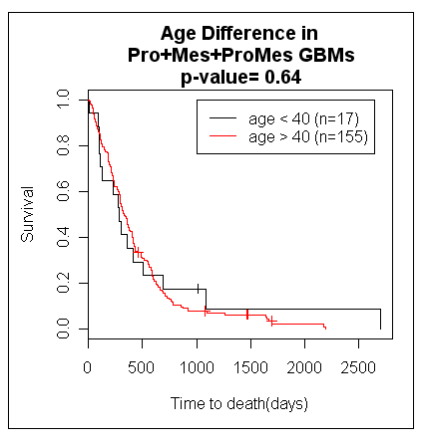
**Kaplan-Meier analysis of GBM patient survival for Pro, Mes, and ProMes GBM patients according to age**. GBM patients suffering from Pro, Mes, and ProMes tumours do not survive any better if younger than age 40 than identically gene expression-classified GBM patients older than the age of 40.

**Figure 4:**
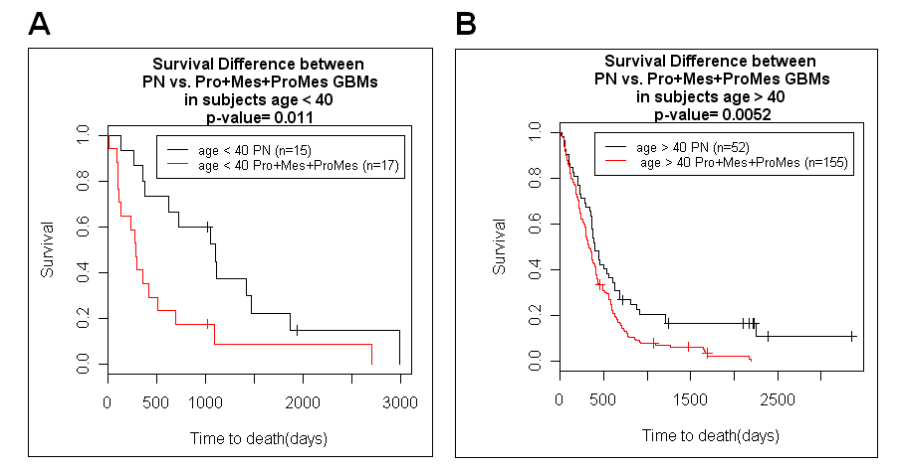
**Kaplan-Meier analysis of GBM patient survival partitioned by age and predictive expression signature**. A. GBM patients younger than age 40 partitioned by PN status versus non-PN status. Younger patients with PN GBMs survive longer than fellow younger patients with non-PN GBMs. PN GBM patients age < 40 survival (mean = 3.0 years, median = 2.9 years) vs. non-PN GBM patients age < 40 survival (mean = 1.4 years, median = 0.8 years). B. GBM patients older than age 40 partitioned by PN status versus non-PN status. Older patients with PN GBMs survive longer than fellow older patients with non-PN GBMs. PN GBM patients age > 40 survival (mean = 1.9 years, median = 1.1 years) vs. non-PN GBM patients age > 40 survival (mean = 1.2 years, median = 0.9 years).

In accordance with previous analyses, patients with PN GBMs (n = 77) have substantially longer survival than patients with non-PN GBMs (Pro, Mes, or ProMes) (n = 190) (P-value = 4.9e^-6^) (Fig. 2A). Patients with PN GBMs have an average survival of 2.2 years (median = 1.4 years) while the patients with non-PN GBMs survive on average 1.2 years (median = 0.9 years). We demonstrate that virtually all of the age effect observed in GBM patients is due to the increased likelihood of being diagnosed with the PN subtype in patients younger than 40. First, patients were partitioned by age to determine if our dataset has the commonly observed beneficial young age affect. As expected, patients younger than 40 years (n = 32) suffering from GBMs survived longer than those over 40 years of age (n = 207) by about two-fold duration (P-value = 0.044) (Fig. 2B). Patients diagnosed younger than age 40 survived on average 2.1 years (median = 1.3 years), and patients over age 40 survived on average 1.4 years (median = 0.9 years). Next, we stratified the patients based on the subtype of GBM. For the patients diagnosed with non-PN GBMs (Pro, Mes, and ProMes), there was no significant difference in survival between younger patients (n = 17) and older patients (n = 155) (P-value = 0.64) (Fig. 3). Thus, when the tumour type was controlled by gene expression-based molecular type, there was no detectable beneficial effect of younger age. When patients were stratified based on age for all of the patients with PN subtypes, age was not a substantial predictor of survival (P-value = 0.09, data not shown). If we ask whether gene expression based classification is a patient survival predictor within the different age groups, PN GBM subtype remains a strong predictor of longer patient survival. Of patients under 40 years of age at diagnosis, those diagnosed with PN GBMs (n = 15) demonstrate a significantly longer survival duration than those similarly young patients who were diagnosed with non-PN GBMs (n = 17) (P-value = 0.011) (Fig. 4A). Similarly, the beneficial gene expression based classifier was detected in the older group of patients. Patients diagnosed at over the age of 40 with PN GBMs (n = 52) survived significantly longer than age-matched patients over the age of 40 that suffered from non-PN GBMs (n = 155) (P-value = 0.0052) (Fig. 4B). Combined, these data indicate that the predominant cause of the beneficial age affect observed in GBM patients is due to the proportionally higher likelihood of patients younger than 40 to be diagnosed with the PN type GBM relative to the Pro, Mes or ProMes types. In our sample set of the patients younger than 40 years of age, 47% (15/32) were PN, while only 25% (52/207) of the patients older than 40 years of age were diagnosed with PN type GBMs (Fishers exact p value = 0.011). GBMs increase in frequency with older age, and of those patients in our dataset over age 60, 23% have the PN type, which indicates a decreasing chance of developing this subtype of GBM with advancing age.

### Multivariate and Univariate Cox proportional hazards analyses support that the molecular signature status of GBMs demonstrates greater hazard prediction than age

To measure the robustness of the PN and non-PN molecular classifier amongst several additional covariates, multivariate and univariate Cox proportional hazards analyses were performed across the GBM patients where age data were available (N = 239: PN (n = 67), non-PN (n = 172) Tables 2 and 3). The model tested the following covariates: age, glioma gene signature, and expression status of MGMT, VEGF, and EGFR. MGMT, VEGF, and EGFR were added in as individual expression covariates due to prior reports of their possible individual contributions in relation to survival prediction [14,15]. Our analysis indicated that only increased VEGF expression significantly correlated with increasing age (data not shown), while MGMT and EGFR expression levels did not show significant correlations with age. While univariate analysis demonstrated as expected that the 'Age 40 and above' covariate represents a near significant marker for hazard (HR = 1.44, P-value = 0.067), age was not a significant survival predictor in the context of multivariate analysis (HR = 1.20, P-value = 0.39). Within the univariate model, the non-PN classification demonstrates a high hazard ratio (Table 3: HR = 1.94, P-value = 3.15e-5). Within the multivariate model, the most significant hazard covariate for patient outcome was in fact the non-PN molecular subtype (Table 2: HR = 1.93, P-value = 1.4e-4).

Finally, several molecular studies have demonstrated that the aberrant expression of certain genes serve as useful prognostic indicators for patient survival. When the expression status of MGMT, VEGF, and EGFR were compared in the multivariate analysis with our molecular classifier, only MGMT expression demonstrated significant performance in the predictive models (Multivariate HR = 1.46, P-value = 6.6e-3). The higher expression of MGMT associated with poorer survival is consistent with other studies that report the expression of MGMT silenced by promoter hypermethylation enhances GBM patient survival [16]. MGMT silencing may also be responsible for conferring additional survival benefit when complemented by Temozolomide treatment, but this could not be studied in the current analysis [17]. In addition to MGMT, VEGF and EGFR expression levels were studied for possible relationships to poor survival outcomes as indicators of angiogenesis and constitutive signal transduction, respectively. However, from the multivariate results neither VEGF nor EGFR 'activation', as determined by the expression arrays, contributed significantly to hazard (VEGF HR = 1.20, P-value = 0.33; EGFR HR = 0.86, P-value = 0.30). Interestingly, with respect to univariate models, one published univariate Cox proportional hazards study reported that VEGF expression was a significant prognostic indicator for poor survival, while EGFR expression was not [15]. Our univariate analysis confirmed this finding as VEGF expression in the univariate model was the only gene whose expression indicated statistically significant hazard amongst these three genes (HR = 1.35, P-value = 0.028), and EGFR failed to display significant hazard implications in both multivariate and univariate models (Univariate HR = 0.91, P-value = 0.51). These weaker effects from individual gene analyses are likely due to their partial contribution to the overall expression signatures. VEGF is part of the ProMes and Mes expression signatures while EGFR is part of the PN, Pro, and ProMes expression signatures. These data may highlight the complex nature of genetic effects within GBMs which are not derived from a single gene but rather a complex reprogramming of hundreds of genes being dysregulated. Thus, the relative contribution of individual, but important, genes is less than the aggregate set defined by the whole gene expression profile. The multivariate analysis supports this conclusion as the ProNeural versus non-ProNeural subtype of GBM within patients demonstrates itself as the primary favourable survival prognosis indicator.

**Table 2:** Multivariate Cox Proportional Hazards Ratio: Five Covariates

Model Covariates	Covariate	Hazard Ratio	95% CI	P-value
AGE	>= 40	1.20	0.80 – 1.80	0.39
PN status	non-PN	1.93	1.38 – 2.70	1.4e-4
MGMT	ON	1.46	1.11 – 1.92	6.6e-3
VEGF	ON	1.20	0.87 – 1.53	0.33
EGFR	ON	0.86	0.64 – 1.15	0.30

**Table 3:** Univariate Cox Proportional Hazards Ratio: Five Covariates

Model Covariates	Covariate	Hazard Ratio	95% CI	P-value
AGE	>= 40	1.44	0.97 – 2.14	0.067
PN status	non-PN	1.94	1.42 – 2.66	3.1e-5
MGMT	ON	1.27	0.97 – 1.66	0.078
VEGF	ON	1.35	1.03 – 1.76	0.028
EGFR	ON	0.91	0.68 – 1.21	0.51

## Discussion

This study uses available genome-scale gene expression based analyses across glioblastomas to further investigate observed age effects in patient survival and creates a unified dataset for further exploration of gene expression correlates in glioblastomas. We add to the literature 86 additional GBM biopsy gene expression profiles performed on the U133A and U133 Plus 2.0 platforms. We confirm that within histologically defined GBMs, there are robust and repeatedly observed gene expression signature based groups of GBMs, which produces a classification scheme within GBMs. Within this aggregate dataset of 267 GBMs, the three previously defined molecular glioma subgroups were robustly detected as defined by over-expression of a series of related genes: Neurogenesis/HC1A (a.k.a. ProNeural), Mitotic/HC2A (a.k.a. Proliferative), and Extra-Cellular Matrix related/HC2B (a.k.a. Mesenchymal). From the larger dataset, we observe evidence of a new subtype that highly co-expresses genes in both the Pro and Mes groups, which we term "ProMes". Of these four molecularly distinct groups identified, only PN portends a favourable prognosis relative to the other expression based groups.

In this study, we determine that the reason that younger patients diagnosed with GBMs have longer survival durations than older GBM patients is due to the observation that younger patients tend to develop the favourable PN GBM type more commonly relative to older patients. The reason for this observed age effect is not clear at this time, but may have to do with the precursor cell that develops into GBMs changing over time. One could hypothesize that the precursor cell that gives rise to the PN type diminishes in abundance in the CNS with advancing age. However, given that GBM incidence increases greatly with age, the absolute numbers of PN GBMs is actually numerically higher in older patient groups. Thus, we favour a model that the precursor cell type that gives rise to the Pro and Mes types of GBMs are increasingly likely to become neoplastic over time while this effect is not as pronounced within the PN type precursors.

PN type GBMs represent a unique tumour aetiology whose idiopathic molecular mechanisms manifest in survival periods spanning from two to ten years in contrast to ten to fifteen months for the Pro, Mes and ProMes types of GBMs. Historically, the percentage of GBM patients who demonstrate long term survival of 3 years or more has been reported to be approximately 5% [18]. This 5% incidence rate matches well with the observations reported here in which 8% (18/267) of our PN GBM patients are observed to survive 3 years or longer. The identification of the PN subgroup is important for patient management and stratification into small phase II clinical trials for experimental therapeutics as uneven representation of the PN GBM diagnoses would greatly alter observed survival times irrespective of potentially active agents [19]. The identification of the gene expression subtype is clearly more important for patient stratification within clinical trials than age.

It is likely that the use of genome-wide expression based molecular classification will result in less variation in tumour diagnoses and provide more specific guidance to clinicians. The agglomeration of gene expression datasets permits meta-analyses that were insufficiently powered in multiple individual publications. Resources for the sharing of genome-scale expression datasets have been set up at Array Express, Gene Expression Omnibus, and Celsius [20,21]. Critical to the sharing of microarray data is providing raw microarray data as opposed to processed data. In order to facilitate this sharing, the NIH Neuroscience Microarray Consortium has established Celsius, which is a community resource of CEL (image) files performed on the Affymetrix platform for public distribution using programmatic tools. At the writing of this manuscript, the Celsius database contains CEL files from human experiments performed on U95Av2 arrays (n = 5 006), U133A arrays (n = 13 818), and U133 Plus 2.0 arrays (n = 10 376). To fully capture and leverage the value of microarray expression data, a greater commitment must be made to capture and share clinical covariates and raw expression data. For instance, in this study of 267 glioblastomas, a total of 433 GBM CEL files were initially identified across the U95Av2, U133A, and U133 Plus 2.0 platforms. Of these, thirty-six percent (160/433) were immediately removed from this study due to a lack of any clinical data. Additionally, not all microarray CEL files or their matched clinical data points are systematically retrievable.

Amongst the samples gathered at UCLA, we simultaneously gathered additional covariates such as extent of surgical resection, Karnofsky Performance Scores (KPS), lesion locations, and MRI scans. We have begun to make all of these data available through a web interface in order to promote data sharing and exploration of gene expression differences in gliomas. All of the data added here are deposited in Gene Expression Omnibus (GEO), and can be explored at our real-time survival-synchronized search engine "Probeset Analyzer" [22].

### Clinical Decision Impact and Improved Public Disclosure

In large academic hospitals, tumours come from a wide variety of patients from across different cities, states, or countries. In contrast, local hospitals treat their regional constituencies. The potential for demographically-biased patient populations and biased tumour subsets is a possibility. These trends can reinforce particular treatment strategies at local institutions over time. For example, if patients from a community highly populated by retirees (e.g. southern Florida) presented with a GBM, clinicians would be apt to predict that these older patients would likely succumb to their malignancies within one year. Current treatment for patients diagnosed with high grade gliomas consists of surgical resection followed by toxic and expensive therapy schedules that are minimally effective. But if these elderly patients were suffering from a PN tumour, they would have a high likelihood for surviving at least two to three years or longer. These patients could then be distinguished from patients who otherwise present identically under the microscope or according to their patient biographical sketch. This would permit time to enrol in potentially beneficial clinical trials. Thus, if grade and age alone were considered for prognosis, these factors would lead clinicians to prescribe unnecessary treatments due to trends reinforced by regional sampling biases.

## Conclusion

We provide an explanation for why younger patients diagnosed with GBM have longer life expectancies than older patients using accumulated whole-genome microarray expression data and clinical variables. We have discovered the reason that younger patients tend to survive longer is because they are more likely to present with PN gene signature tumours relative to the more common and aggressive Pro, Mes, and ProMes GBM types. The PN molecular classification predicts an enhanced survival performance by at least two to ten years irrespective of age when tested against age-matched, Pro, Mes, or ProMes molecularly-classified tumours. The application of Cox proportional hazards studies have also confirmed that having a non-PN tumour was the most statistically significant factor in predicting precipitous short survival over age by two orders of magnitude. These data lend more evidence to the clinical reality that high-grade gliomas can be molecularly distinct tumours. Therefore, different tumours with distinct aetiologies should be differentially segregated in terms of their treatment regimens and especially their clinical trial assignments. The benefits for clinicians would be a reduction in the heterogeneous admixture of genetic background for treatment cohorts and a potential reduction in the percentage of non-responders for molecularly-targeted investigational new drugs. Patients accurately classified and characterized for the biology of their tumour may potentially benefit from a variety of molecularly-targeted treatments as the inclusion criteria for clinical trials become simultaneously based on molecular signatures.

## Methods

### Microarray and Clinical Data Collection

Clinical data including histopathology, age, sex, and survival time from diagnosis were retrieved from 181 glioblastomas which have been reported within previous studies between 2003 and 2006 (Table 1) and for which CEL files (Affymetrix, Santa Clara, CA) were available from the authors. In addition, we collected 86 new patient-unique tumour biopsies from the UCLA Neuro-oncology Program (n = 55) and the Barrow Neurological Institute (n = 31) for a grand total of 267 glioblastomas. Newly acquired tumours were collected through institutional review board approved protocols and assigned WHO grades at UCLA Neuropathology or Barrow Neuropathology by PSM. Time of survival (days), sex, and, age were collected where available [see Additional file 1]. Patient age at the time of diagnosis was available for 239 patients and ranged from 18 to 86 years. Sex of the individual was available for those 239 patients (151 males and 88 females).

### Microarray Experimentation

Total RNA was purified from fresh frozen tumour biopsies and visually inspected for tumour content using Qiagen RNAeasy columns and standard manufacturer's protocols. Labelled one round cRNA was generated using kits (GeneChip One-Cycle Target Labelling and Control Reagent) from Affymetrix. cRNA was quantified and 15 micrograms were hybridized to U133A and U133 Plus 2.0 arrays at the UCLA DNA Microarray Facility using standard protocols recommended by the manufacturer [see Additional file 2]. All newly generated CEL files were deposited into the Celsius microarray database and this system was used to normalize relative to other microarrays of the same Affymetrix platform using RMA with default settings from the Bioconductor R library [20,21,23,24].

### Combination of microarray data

The Celsius microarray database, which houses over 20 000 human CEL files on various array iterations, was used to quantify and normalize the three microarray platform datasets with comparison groups of 50 random samples selected from each respective platform for RMA normalization and quantification using default parameters. Only probesets from the U133A portion of the Freije *et al*. paper that are retained or map to the same gene were analyzed from each tumour type. All microarray CEL files analyzed in this study in conjunction with their clinical covariates are accessible from the Gene Expression Omnibus (GEO) (Series Accession number: GSE13041).

### Sample membership by HC Classification Gene Voting

The Hierarchical Clustering (HC) classification for each glioma was determined by the gene voting strategy as described previously [8]. Briefly, the mean value of each probeset was evaluated from all samples within each of the three microarray platforms U95Av2, U133A, and U133 Plus 2.0 separately. Second, the probesets from each sample were assigned "yes" or "no" votes if that probeset's value was above or below the aforementioned probeset mean of its platform. Third, the "yes" or "no" votes of each probeset from the 377 probesets contained within the U133A portion of the 595 HC probeset classifier (which was based on U133A and U133B data) were tallied and used to categorize every glioma into one of three HC molecular groups. All of the 377 probesets from the 377 used for classification were on the U133A and the U133 Plus 2.0 array types, and 177 of the probesets were able to be matched to the same genes from the older U95Av2 arrays. For probesets that were unavailable on the U95Av2 arrays, the mean value of the probesets on the U133A and U133 Plus 2.0 arrays were calculated and intercalated for the missing row values of the U95Av2 samples strictly for visualization purposes and not gene voting. Lastly, each tumour was voted into one of three HC groups based on the highest vote tally: 1A vote = (tally of 1A probesets above the mean)/(total 1A probesets); 2A vote = (tally of 2A probesets above the mean)/(total 2A probesets); 2B vote = (tally of 2B probesets above the mean)/(total 2B probesets). A few gliomas appeared to vote almost equally well into both the 2A and 2B categories and are defined as "2A2B", which is defined where the highest vote category must be a 2A or 2B and the second highest vote must be 2B or 2A, respectively with at least a 33% vote. The lists of probesets used for voting each tumour across each of the Affymetrix platforms is available [see Additional file 3].

### Kaplan-Meier and Cox Regression Analysis

Kaplan-Meier survival plots and Cox proportional hazard regression analyses were implemented in R version 2.5.0 using the "survival" library. Survival data and vital status for UCLA samples were based on time in days elapsed from surgical resection to the date of death up to May 1, 2006. The full R code is available and documented [see Additional file 4].

### MGMT, VEGFA, EGFR Expression State Determination

Expression states for the MGMT, VEGFA and EGFR probesets were called "ON" or "OFF" based on whether their probesets' expression levels were above or below their respective mean within each platform. The probesets employed for the genes from each platform are listed [see Additional file 3].

## Abbreviations

GBM: Glioblastoma; WHO: World Health Organization; HC1A/PN: Hierarchical Cluster group 1A (Neurogenesis)/ProNeural; HC2A/Pro: Hierarchical Cluster group 2A (Mitotic)/Proliferative; HC2A2B/ProMes: Hierarchical Cluster group 2A2B/Proliferative Mesenchymal; HC2B/Mes: Hierarchical Cluster group 2B (Extra-Cellular Matrix)/Mesenchymal; MGMT: *O-6-methylguanine-DNA-methyltransferase *(GeneID:4255); VEGFA: *vascular endothelial growth factor A *(GeneID: 7422); EGFR: *epidermal growth factor receptor *(GeneID: 1956), Temozolomide (Brand Name: Temodar^®^).

## Competing interests

Financial competing interests:

The authors declare that they have no competing interests.

Non-financial competing interests:

The authors declare that they have no competing interests.

## Authors' contributions

YL carried out the public and newly obtained data acquisition, analyzed the microarray expression data, performed the gene voting, identified the 2A2B/ProMes group, performed all statistical analyses (except the positive correlation between age and VEGF expression), and drafted the manuscript. ACS participated actively in revising the manuscript, obtained a portion of the newly acquired glioma biopsies, and collected the clinical covariates for those glioma biopsies. TFC participated in the design of the study, collected the clinical covariates for the entire UCLA subset of the glioma biopsies, proposed the VEGF and EGFR expression studies, and helped revise the manuscript. AL participated in the design of the study, collected additional clinical covariates for the entire UCLA subset of the glioma biopsies, proposed the MGMT expression studies, and screened all of the UCLA clinical covariates. JD participated in the design of the statistical analyses, provided substantive assistance for interpretation of the statistical calculations, and performed the correlation analysis. HKF performed the tissue biopsy collection, micro-dissection, and electronic patient identification assignment and confirmation for all the newly acquired UCLA tumour biopsies. LML collected the newly acquired UCLA tumour biopsies, participated in the conception of the manuscript, and helped revise the manuscript. SH was involved in the initial conception, design, of the statistical analyses. PSM participated in the design of the study, performed histopathological grading on the public and newly acquired UCLA tumour biopsies, and provided input and revisions on the manuscript. SFN conceived of the study and its design, supervised all analyses, and led the drafting and editing of the manuscript. All authors read and approved the final manuscript.

## Pre-publication history

The pre-publication history for this paper can be accessed here:



## Supplementary Material

Additional file 1 **Tumour sample clinical covariates meta-data.** e.g. Time of survival (days), sex, and age where available. Click here for file

Additional file 2 **Standard protocol recommended by Affymetrix used at UCLA DNA Microarray Facility.** Click here for file

Additional file 3 **List of Affymetrix probesets used for voting each tumour across each of the Affymetrix platforms.** Click here for file

Additional file 4 **The full R code used for Kaplan-Meier survival curves, Multivariate/Univariate Cox proportional Hazards models, and correlations between age and gene expression.** Click here for file
