# Supplementary material for: Gene expression analysis of glioblastomas identifies the major molecular basis for the prognostic benefit of younger age
Source: BMC Med Genomics. 2008 Oct 21;1:52. doi: 10.1186/1755-8794-1-52 (PMC2596165; doi:10.1186/1755-8794-1-52)
Supplement: Additional file 2 — Standard protocol recommended by Affymetrix used at UCLA DNA Microarray Facility. [file 1755-8794-1-52-S2.doc]

Supplementary_Information_section_S2

Labeled one round cRNA was generated using kits (GeneChip One-Cycle Target Labeling and Control Reagent) from Affymetrix. cRNA was quantified and 15 micrograms were hybridized to U133A and U133 Plus 2.0 arrays at the UCLA DNA Microarray Facility (http://microarray.genetics.ucla.edu/) using standard protocols recommended by the manufacturer.

Briefly, all RNA samples were isolated as previously described and analyzed for concentration by Nanodrop (NanoDrop Technologies, Wilmington, DE) and total RNA integrity with Agilent 2100 Bioanalyzer (Agilent Technologies, Palo Alto, CA) [11]. Samples displayed 28s/18s ratios over 1.5 with no evidence of degradation. Quality Control workflow steps via GCOS v1.4 (Affymetrix, Santa Clara, Ca) were performed on the targets produced and the hybridizations that were applied. Hybridization and Poly-A controls all revealed trends within expected parameters and 3'/5' ratios for Actin & GAPDH well below 3 (mean Beta Actin 3'/5' = 1.53 & mean GAPDH 3'/5' = 1.02). GCOS v1.4 Expression Reports also revealed expected Call percentages (Present: 48% to 62%; Absent: 36% to 49%; Marginal: 1.5% to 1.9%). All arrays were within 1.5 fold of each other in overall intensity, and array images were visually inspected for surface defects.
