# Supplementary material for: Gene expression analysis of glioblastomas identifies the major molecular basis for the prognostic benefit of younger age
Source: BMC Med Genomics. 2008 Oct 21;1:52. doi: 10.1186/1755-8794-1-52 (PMC2596165; doi:10.1186/1755-8794-1-52)
Supplement: Additional file 4 — The full R code used for Kaplan-Meier survival curves, Multivariate/Univariate Cox proportional Hazards models, and correlations between age and gene expression. [file 1755-8794-1-52-S4.doc]

Supplementary_Information_section_S4

source: Supplementary_Information_section_S1.xls

#Figure_2A_Survival_Difference_between_PN_vs_non-PN_GBMs

#file: Fig2A_N(267)_PN(77)_nPN(190).txt

setwd("enter_path")

library(survival)

rm(list=ls())

dat2a=read.table("Fig2A_N(267)_PN(77)_nPN(190).txt",sep="\t", header=T)

attach(dat2a)

names(dat2a)

censor2a <- ifelse(STATUS..A.0.D.1.=="DECEASED",1,0) #(logic, true, false)

label2a=factor(GRP..2.yes.1A..3.no.1A.)

fit.diff2a <- survdiff(Surv(TTS,censor2a)~label2a) #can enumerate TTS as 'TTS2a'

chisq2a <-signif(1-pchisq(fit.diff2a$chisq,length(levels(factor(label2a)))-1), 2)

fit2a <- survfit(Surv(TTS,censor2a)~label2a, conf.type="log-log")

no.lines2a <- length(levels(factor(label2a)))+1 #can enumerate TTS as 'TTS2a'

plot(fit2a, conf.int=F, xlab="Time to death(days)",col=c(1:2),ylab="Survival",

main=paste("Survival Difference between\n PN vs. Pro+Mes+ProMes GBMs\n p-value=",chisq2a))

legend(500, 1, lty=1, col=1:2, c("PN (n=77)","Pro+Mes+ProMes (n=190)"))

#p-value: 4.9e-06

--

#Figure_2B_Age_Difference_in_GBMs

#file: Fig2B_N(239)_l40(32)_m40(207).txt

setwd("enter_path")

library(survival)

rm(list=ls())

dat2b=read.table("Fig2B_N(239)_l40(32)_m40(207).txt",sep="\t", header=T)

attach(dat2b)

names(dat2b)

censor2b <- ifelse(STATUS..A.0.D.1.=="DECEASED",1,0) #(logic, true, false)

label2b=factor(AGE..4.39..5.40.)

fit.diff2b <- survdiff(Surv(TTS,censor2b)~label2b) #can enumerate TTS as 'TTS2b'

chisq2b <-signif(1-pchisq(fit.diff2b$chisq,length(levels(factor(label2b)))-1), 2)

fit2b <- survfit(Surv(TTS,censor2b)~label2b, conf.type="log-log")

no.lines2b <- length(levels(factor(label2b)))+1 #can enumerate TTS as 'TTS2b'

plot(fit2b, conf.int=F, xlab="Time to death(days)",col=c(1:2),ylab="Survival",

main=paste("Age Difference in GBMs\n p-value=",chisq2b))

legend(1000, 1, lty=1, col=1:2, c("age < 40 (n=32)","age > 40 (n=207)"))

# p-value: 0.044

--

#Figure_3_Age_Difference_in_Pro_Mes_ProMes_GBMs

#file: Fig3_nPN_l40(17)_m40(155).txt

setwd("enter_path")

library(survival)

rm(list=ls())

dat3=read.table("Fig3_nPN_l40(17)_m40(155).txt",sep="\t", header=T)

attach(dat3)

names(dat3)

censor3 <- ifelse(STATUS..A.0.D.1.=="DECEASED",1,0) #(logic, true, false)

label3=factor(AGE..4.39..5.40.)

fit.diff3 <- survdiff(Surv(TTS,censor3)~label3) #can enumerate TTS as 'TTS3'

chisq3 <-signif(1-pchisq(fit.diff3$chisq,length(levels(factor(label3)))-1), 2)

fit3 <- survfit(Surv(TTS,censor3)~label3, conf.type="log-log")

no.lines3 <- length(levels(factor(label3)))+1 #can enumerate TTS as 'TTS3'

plot(fit3, conf.int=F, xlab="Time to death(days)",col=c(1:2),ylab="Survival",

main=paste("Age Difference in\n Pro+Mes+ProMes GBMs\n p-value=",chisq3))

legend(1000, 1, lty=1, col=1:2, c("age < 40 (n=17)","age > 40 (n=155)"))

# p-value: 0.64

--

#Figure_4A_Survival_Difference_in_PN_vs_Pro_Mes_ProMes_GBMs_age_less_40

#file: Fig4A_l40_PN(15)_nPN(17).txt

setwd("enter_path")

library(survival)

rm(list=ls())

dat4a=read.table("Fig4A_l40_PN(15)_nPN(17).txt",sep="\t", header=T)

attach(dat4a)

names(dat4a)

censor4a <- ifelse(STATUS..A.0.D.1.=="DECEASED",1,0) #(logic, true, false)

label4a=factor(GRP..2.yes.1A..3.no.1A.)

fit.diff4a <- survdiff(Surv(TTS,censor4a)~label4a) #can enumerate TTS as 'TTS4a'

chisq4a <-signif(1-pchisq(fit.diff4a$chisq,length(levels(factor(label4a)))-1), 2)

fit4a <- survfit(Surv(TTS,censor4a)~label4a, conf.type="log-log")

no.lines4a <- length(levels(factor(label4a)))+1 #can enumerate TTS as 'TTS4a'

plot(fit4a, conf.int=F, xlab="Time to death(days)",col=c(1:2),ylab="Survival")

title(main=paste("Survival Difference between\n PN vs. Pro+Mes+ProMes GBMs\nin subjects age < 40\n p-value=",chisq4a), col="black", cex.main=1, font=3)

legend(500, 1, lty=1, col=1:2, cex=0.8, c("age < 40 PN (n=15)","age < 40 Pro+Mes+ProMes (n=17)"))

# p-value: 0.011

--

#Figure_4B_Survival_Difference_in_PN_vs_Pro_Mes_ProMes_GBMs_age_more_40

#file: Fig4B_m40_PN(52)_nPN(155).txt

setwd("enter_path")

library(survival)

rm(list=ls())

dat4b=read.table("Fig4B_m40_PN(52)_nPN(155).txt",sep="\t", header=T)

attach(dat4b)

names(dat4b)

censor4b <- ifelse(STATUS..A.0.D.1.=="DECEASED",1,0) #(logic, true, false)

label4b=factor(GRP..2.yes.1A..3.no.1A.)

fit.diff4b <- survdiff(Surv(TTS,censor4b)~label4b) #can enumerate TTS as 'TTS4b'

chisq4b <-signif(1-pchisq(fit.diff4b$chisq,length(levels(factor(label4b)))-1), 2)

fit4b <- survfit(Surv(TTS,censor4b)~label4b, conf.type="log-log")

no.lines4b <- length(levels(factor(label4b)))+1 #can enumerate TTS as 'TTS4b'

plot(fit4b, conf.int=F, xlab="Time to death(days)",col=c(1:2),ylab="Survival")

title(main=paste("Survival Difference between\n PN vs. Pro+Mes+ProMes GBMs\nin subjects age > 40\n p-value=",chisq4b), col="black", cex.main=1, font=3)

legend(400, 1, lty=1, col=1:2, cex=0.8, c("age > 40 PN (n=52)"," age > 40 Pro+Mes+ProMes (n=155)"))

# p-value: 5.2e-3

--

#Table_2_Multivariate_Cox_Proportional_Hazards_Ratio

#Table_3_Univariate_Cox_Proportional_Hazards_Ratio

#file: N(239)_PN(67)_nPN(172)_coxph.txt

setwd("enter_path")

library(survival)

#### Multivariate Regression Analysis (coxph)

rm(list=ls())

dat1=read.table("N(239)_PN(67)_nPN(172)_coxph.txt", sep="\t", header=T, row.names=1)

colnames(dat1)=c("TTS", "STATUS", "AGE", "GRP", "MGMT", "VEGF", "EGFR")

dat1$STATUS=ifelse(dat1$STATUS=="DECEASED", 1,0)

dim(dat1);

dat1[1:3,]

library(survival)

apply(dat1[,-1],2,table)

cox1m=coxph( Surv(TTS, STATUS) ~ as.factor(AGE) + as.factor(GRP) + as.factor(MGMT) + as.factor(VEGF) + as.factor(EGFR), dat1)

summary(cox1m)

#### Univariate Regression Analysis (coxph)

cox1u=coxph( Surv(TTS, STATUS) ~ as.factor(AGE), dat1); summary(cox1u)

cox1u=coxph( Surv(TTS, STATUS) ~ as.factor(GRP), dat1); summary(cox1u)

cox1u=coxph( Surv(TTS, STATUS) ~ as.factor(MGMT), dat1); summary(cox1u)

cox1u=coxph( Surv(TTS, STATUS) ~ as.factor(VEGF), dat1); summary(cox1u)

cox1u=coxph( Surv(TTS, STATUS) ~ as.factor(EGFR), dat1); summary(cox1u)

#### Correlation of age group with gene expression on/off

setwd("enter_path")

age4=read.table("age_gene_corr.txt", sep="\t", header=T, nrows=32); dim(age4); head(age4);

age5=read.table("age_gene_corr.txt", sep="\t", header=T, skip=35, nrows=207); dim(age5); head(age5);

old=c( rep(0, dim(age4)[1]),rep(1, dim(age5)[1]) ); length(old);

MGMT=c(age4[,6], age5[,6]); MGMT=MGMT-min(MGMT); table(MGMT);

VEGF=c(age4[,7], age5[,7]); VEGF=VEGF-min(VEGF); table(VEGF);

EGFR=c(age4[,8], age5[,8]); EGFR=EGFR-min(EGFR); table(EGFR);

### Correlation tests and their p-values

cor.test(old, MGMT); p-value = 0.2767

Pearson's product-moment correlation

data: old and MGMT

t = -1.0902, df = 237, p-value = 0.2767

alternative hypothesis: true correlation is not equal to 0

95 percent confidence interval: -0.19577832 0.05676568

sample estimates: cor -0.07063817

cor.test(old, VEGF); p-value = 0.01841

Pearson's product-moment correlation

data: old and VEGF

t = 2.3737, df = 237, p-value = 0.01841

alternative hypothesis: true correlation is not equal to 0

95 percent confidence interval: 0.02599694 0.27398610

sample estimates: cor 0.1523893

cor.test(old, EGFR); p-value = 0.6436

Pearson's product-moment correlation

data: old and EGFR

t = -0.4633, df = 237, p-value = 0.6436

alternative hypothesis: true correlation is not equal to 0

95 percent confidence interval: -0.15637904 0.09718507

sample estimates: cor -0.03008093
